# Understanding the effect of measurement time on drug characterization

**DOI:** 10.1371/journal.pone.0233031

**Published:** 2020-05-14

**Authors:** Hope Murphy, Gabriel McCarthy, Hana M. Dobrovolny

**Affiliations:** Department of Physics & Astronomy, Texas Christian University, Fort Worth, TX, United States of America; Flinders University of South Australia, AUSTRALIA

## Abstract

In order to determine correct dosage of chemotherapy drugs, the effect of the drug must be properly quantified. There are two important values that characterize the effect of the drug: *ε*_max_ is the maximum possible effect of a drug, and IC_50_ is the drug concentration where the effect diminishes by half. There is currently a problem with the way these values are measured because they are time-dependent measurements. We use mathematical models to determine how the *ε*_max_ and IC_50_ values depend on measurement time and model choice. Seven ordinary differential equation models (ODE) are used for the mathematical analysis; the exponential, Mendelsohn, logistic, linear, surface, Bertalanffy, and Gompertz models. We use the models to simulate tumor growth in the presence and absence of treatment with a known IC_50_ and *ε*_max_. Using traditional methods, we then calculate the IC_50_ and *ε*_max_ values over fifty days to show the time-dependence of these values for all seven mathematical models. The general trend found is that the measured IC_50_ value decreases and the measured *ε*_max_ increases with increasing measurement day for most mathematical models. Unfortunately, the measured values of IC_50_ and *ε*_max_ rarely matched the values used to generate the data. Our results show that there is no optimal measurement time since models predict that IC_50_ estimates become more accurate at later measurement times while *ε*_max_ is more accurate at early measurement times.

## Introduction

In the 21st century it is expected that cancer will be the leading cause of death worldwide [1]. The first or second leading cause of death for people below the age of 70 is cancer in 91 of 172 countries, including the US where cancer is the leading cause of premature mortality [1]. Diagnosis and treatment of cancer can be medically and technically complex [2]. While there are many new therapies being developed for cancer [3–5], chemotherapy is still a staple of cancer treatment [6].

Determining the correct dose of chemotherapy is a difficult process that has been used for years [7]. This process involves two important quantities that characterize the effect of the drug. These quantities are the maximum possible effect of a drug (*ε*_max_), and the drug concentration where the effect diminishes by half (IC_50_). The current method to find drug effect uses dose-response curves. Unfortunately, the measured IC_50_, as well as measured *ε*_max_, depend on the exact day that is chosen to make the measurement; this effect has been observed in both modeling studies [8–12] and in experimental studies [9, 13–17]. Cells are grown in the presence of various concentrations of drug and measurement of the number of cells on a particular day is used to determine the relative drug effect and the dose-response curve. The shape of the dose-response curve changes depending on what day the values are measured. This inaccuracy leads to a time-dependent bias. Two possible reasons for this error are due to the initial exponential growth and drug effect stabilization delays [9]. How this inaccuracy affects IC_50_ and *ε*_max_ estimates can be better understood through the use of mathematical modeling.

Mathematical models have been widely used in the study of cancer treatment going back to the 1960s when models were developed to predict the growth of tumors [18–21]. More recently, models are being used to optimize [22, 23] or even personalize [24–26] treatment regimens for patients. While mathematical models of tumor growth have become increasingly complex [27, 28], simpler ordinary differential equation (ODE) models can still help provide insight into cancer dynamics. Such ODE models have been used to make predictions about the effectiveness of cancer treatments [29, 30], including combination therapies [31, 32] and help improve the way efficacy is measured [31, 33].

In this paper, we use ODE mathematical models to examine the time-dependence of IC_50_ and *ε*_max_ estimates. We find that *ε*_max_ and IC_50_ values vary largely as a function of the measurement time. This is problematic because it can change the treatment dose estimate for patients. We also complete a sensitivity analysis to understand how model parameters affect the IC_50_ and *ε*_max_ estimates. We find that the estimated values of IC_50_ and *ε*_max_ are model-dependent with some estimates also being highly correlated to model parameters. However, the trend for the majority of the models is that *ε*_max_ increases with increasing measurement time while IC_50_ decreases with increasing measurement time.

## Materials and methods

### Mathematical models

In this paper, we use seven common ODE models of tumor growth. The models predict the growth of a tumor by describing the change in tumor volume, *V*, over time. Parameters *a*, *b*, and *c* can be adjusted to describe a particular data set. Equations for the models are in Table 1.

**Table 1:** ODE tumor growth models.

Model	Equation
Exponential	$\dot{V}=aV$
Mendelsohn	$\dot{V}=aV^{b}$
Logistic	$\dot{V}=aV(1-\frac{V}{b})$
Linear	$\dot{V}=\frac{aV}{\left(V+b\right)}$
Surface	$\dot{V}=\frac{aV}{{\left(V+b\right)}^{\frac{1}{3}}}$
Gompertz	$\dot{V}=aV\ln\frac{b}{\left(V+c\right)}$
Bertalanffy	$\dot{V}=aV^{\frac{2}{3}}-bV$

#### Exponential

When tumors first begin to form, two daughter cells are created each time the cell divides. This makes the exponential model a good description for tumors when they first begin to grow [34]. The growth of the tumor is proportional to the volume of the tumor, where *a* is the growth rate of the tumor. The exponential model is inaccurate after short periods of time as a result of cells having limited resources [35].

#### Mendelsohn

Mendelsohn created a generalization of the exponential model [36]. Growth of the tumor is proportional to volume raised to some power, *b*. This model reduces to an exponential equation when *b* equals 1 [35].

#### Logistic

Pierre Francois Verhulst developed the logistic (or Pearl-Verhulst) equation in 1838 [37]. The logistic equation can explain the decrease in tumor growth as the tumor gets larger by assuming that the growth rate (*a*) reduces linearly with size until it equals zero at the carrying capacity (*b*), with the resulting sigmoid growth curve being symmetric.

#### Linear

This model predicts that growth of the tumor starts off exponentially and becomes linear growth at later times. This means that the radius grows at a rate of $V^{\frac{1}{3}}$. The model was used to model growth of bacterial colonies in a culture, and is a good model to replicate the growth of tumors in a dish [21].

#### Surface

Our formulation again assumes exponential growth (*a*) at early times with surface growth taking over at longer times. The model does not work for growth over extended periods of time because it doesn’t account for the decline in growth rate for cancerous cells as the tumor gets large [38].

#### Bertalanffy

Ludwig Bertalanffy developed the Bertalanffy model to describe the growth of organisms [39]. The model predicts a sigmoid shape growth curve with a decrease of tumor volume due to cell death, proportional to the volume of the tumor.

#### Gompertz

The Gompertz model was developed in 1825 by Benjamin Gompertz to describe human mortality curves [40]. The cells in a tumor are not all dividing but the cells that divide are dividing at a rate that is similar to early stage growth. The model is similar to the logistic model, but the sigmoidal curve is not symmetric at the point of inflection.

The mathematical models are simulated using the parameter values from Fig. 3 in [41], presented in Table 2. The parameter values were estimated using data from Worschech et al. worschech09 of a GI-101A xenograft in nude mice (Fig 1A of [42], control data).

**Table 2:** Parameters for the seven ODE models. The parameter values are from Fig. 3 in [41].

Model	a	b	c
Exponential	0.0246 /d		
Mendelsohn	0.105 /d	0.785	
Logistic	0.0295 /d	6920 mm^3^	
Linear	132 mm^3^/d	4300 mm^3^	
Surface	0.291 mm/d	708 mm^3^	
Gompertz	0.0919 /d	15500 mm^3^	10700 mm^3^
Bertalanffy	0.306 mm/d	0.0119 /d	

### Implementing drug effect

Since we are investigating measurement of drug efficacy parameters, we need to incorporate drug effect in our model. We use the drug efficacy, *ϵ*, given by

$$\begin{array}{c} \epsilon =\frac{\varepsilon_{\text{max}}D^{\gamma }}{D^{\gamma }+{\text{IC}}_{50}^{\gamma }}, \end{array}$$ (1)

where *D* is the drug concentration, *ε*_max_ is the maximum possible effect of a drug, IC_50_ is the drug concentration where the effect diminishes by half, and *γ* is the Hill coefficient. The Hill coefficient is a measure of binding cooperativity of the drug; a Hill coefficient greater than one means that drug binding at one site makes it easier for drugs to bind at other sites. We assume that the drug is given on day one and a constant dose of drug is applied to the cells. *ϵ* gives the relative reduction in a particular parameter where *ϵ* = 0 means that there is no effect and *ϵ* = 1 means 100% reduction. For example, if we assume that the drug decreases *a*, we multiply *a* by (1 − *ϵ*) to represent the effect of the drug in the model. For simulations, we assume that both *ε*_max_ and IC_50_ are 1. For *ε*_max_, this means we assume that we have a perfectly effective drug. For IC_50_, this assumption is equivalent to expressing drug concentrations relative to IC_50_. For most of our simulations, we also assume that *γ* = 1. In this study, we do not model any specific drug, but rather apply the drug to the different parameters in each of the models. In some cases, this results in simulation of a known drug [43–45], but in other cases, this is a theoretical exercise without replicating the effects of a specific drug.

### Estimating *ε*_max_ and IC_50_

We simulate the growth of cancer cells (GI-101A parameters) in the presence of different concentrations of drug and measure the relative drug effect,

$$\begin{array}{c} R=1-\frac{V_{d}\left(t\right)}{V_{\text{nd}}\left(t\right)}, \end{array}$$ (2)

where *V*_d_(*t*) is the volume of the tumor remaining at time *t* after drugs are applied to the cells and *V*_nd_(*t*) is the volume of the tumor at the time *t* when no drugs are applied to the cells. A dose-response curve is generated by plotting the relative drug effect vs. log(*D*) measured on a particular day. The dose-response curve is a sigmoidal curve given by

$$\begin{array}{c} R=\frac{\varepsilon_{\text{max}}D}{D+{\text{IC}}_{50}}. \end{array}$$ (3)

Curve fitting of this equation to the dose-response curve is used to estimate *ε*_max_ and IC_50_ for each measurement day. The best fit is determined by minimizing the SSR using the Python Scipy curve_fit function, which fits a sigmoid function to the data.

## Results

### Determining time-dependence of IC_50_ and *ε*_max_

**Fig 1:**
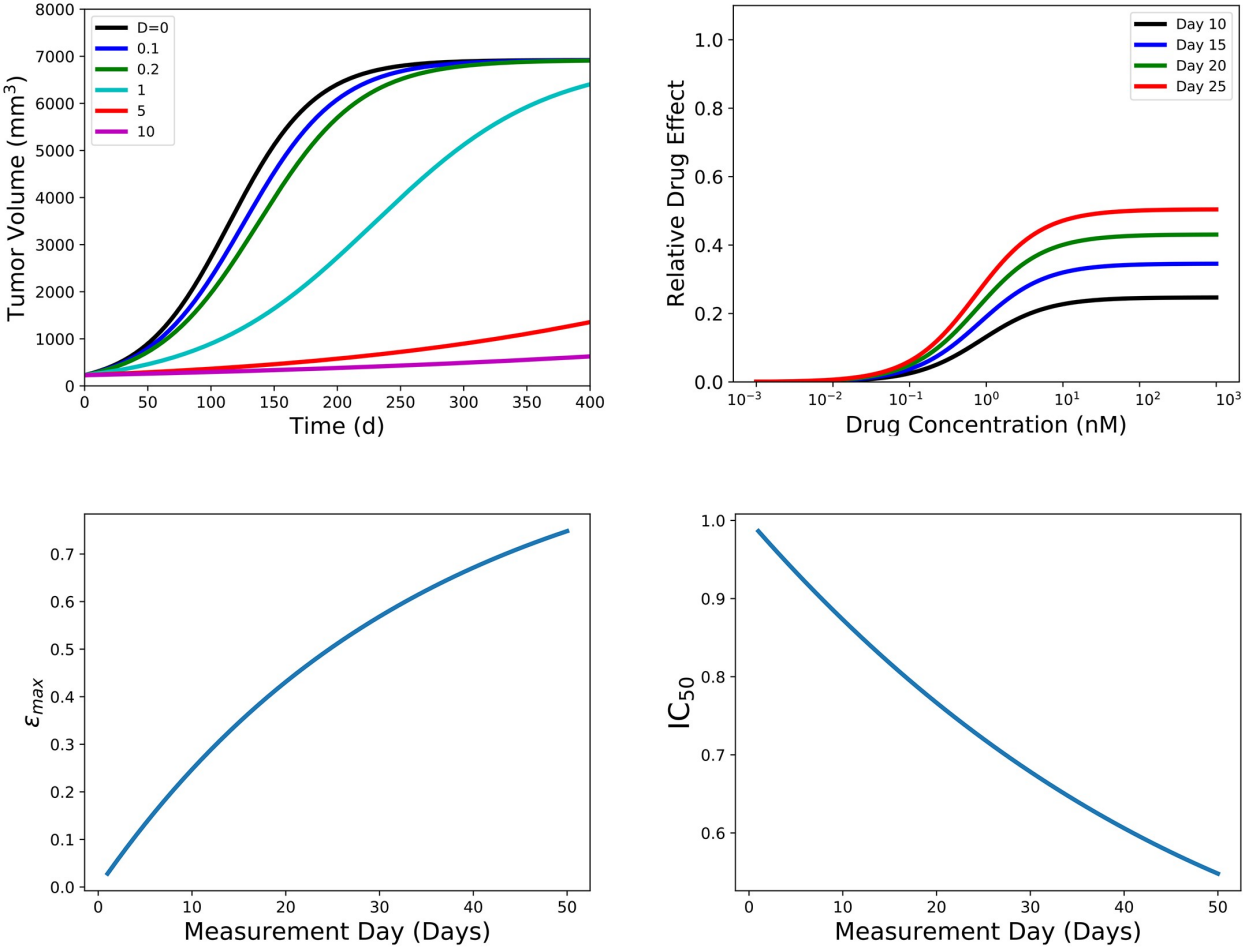
Example of the process used to determine time dependence of *ε*_max_ and IC_50_. (top left) We use the logistic model with a drug that reduces the growth rate to examine how the drug concentration affects the tumor volume. (top right) shows the dose response curves generated for several different measurement days. Individual dose response curves are used to estimate the *ε*_max_ (bottom left) and IC_50_ (bottom right) over 50 measurement days.

We use the logistic model as an example to show how the time-dependence of IC_50_ and *ε*_max_ is calculated; results for the remaining models are in the supplementary material. The graphs in Fig 1 illustrate our process for the logistic model with a drug that reduces growth rate. Fig 1 (top left) demonstrates how drug concentration affects the growth of a tumor by graphing the volume of the tumor as a function of time for several drug concentrations. The corresponding dose-response curves are shown in Fig 1 (top right) for several measurement times. Fig 1 (bottom row) show the measured values of *ε*_max_ (left) and IC_50_ (right) over a range of measurement times. We see that for this model, *ε*_max_ increases with measurement time while IC_50_ decreases with measurement time. Since this is a simulation, we know the expected values of both *ε*_max_ and IC_50_ (both were set to 1); there is no measurement time at which either parameter is correctly estimated. We also see that increasing measurement time brings us closer to the expected value of *ε*_max_, but takes us further from the expected value of IC_50_, so there does not seem to be an optimal measurement time that would give reasonable estimates for both parameters.

**Fig 2:**
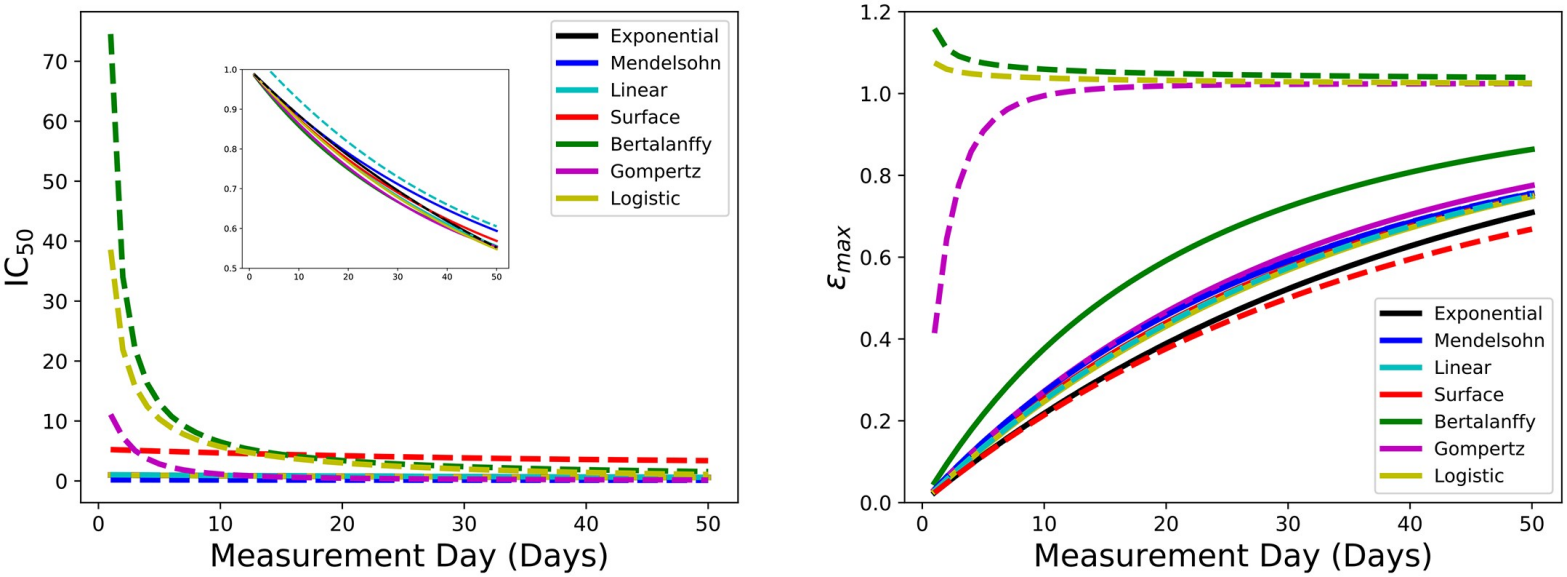
IC_50_ (left) and *ε*_max_ (right) measurement time dependence for all seven models. Solid lines represent the drug effect applied to *a* and dashed lines represent an effect applied to *b*.

Fig 2 shows the IC_50_ and *ε*_max_ measurement time dependence for all the models examined. Fig 2 (left) shows drastic differences in IC_50_ values when measurements are taken on different days. Some models predict that measured IC_50_ can decrease substantially during the first 10 days. The most dramatic change is for the Bertalanffy model with a drug effect applied to *b* where the range of IC_50_ values decreases by 80 times. The logistic model with a drug effect applied to *b* shows a 40-fold change and the Gompertz model with a drug applied to *b* or *c* drops by a factor of 10. All of the other models show a steady decrease in IC_50_ with increasing measurement day, but estimate that IC_50_ is always less than the expected value of 1.

Fig 2 (right) shows *ε*_max_ as a function of measurement day for the different models. In this figure we see that *ε*_max_ generally increases as measurement day increases. The increase in *ε*_max_ is fairly gradual, with a maximum value of 0.9 at measurement day fifty, when the drug acts on *a* for the logistic, exponential, Mendelsohn, linear, surface, Bertalanffy, and Gompertz models. The predicted *ε*_max_ value also has a similar trend when the drug acts on *b* for the Mendelsohn, linear, and surface models. A few models exhibit slightly different behavior. We see that the *ε*_max_ values increase rapidly as measurement day increases and then level off at approximately 1 around day 10 when the drug acts on either *b* or *c* for the Gompertz model. Fig 2 shows that when the drug acts on *b* for the logistic and the Bertalanffy model, the predicted *ε*_max_ values are all above 1 and decrease as measurement day increases. The correct value of *ε*_max_ in this case is 1, so we would hope that the measurement procedure returns this value of *ε*_max_. Fig 2 shows that the current experimental measurement technique almost never returns the correct value of *ε*_max_. The exceptions being the Gompertz, Bertalanffy, and logistic models, although only if the drug is applied to parameter *b*.

### Sensitivity analysis

**Fig 3:**
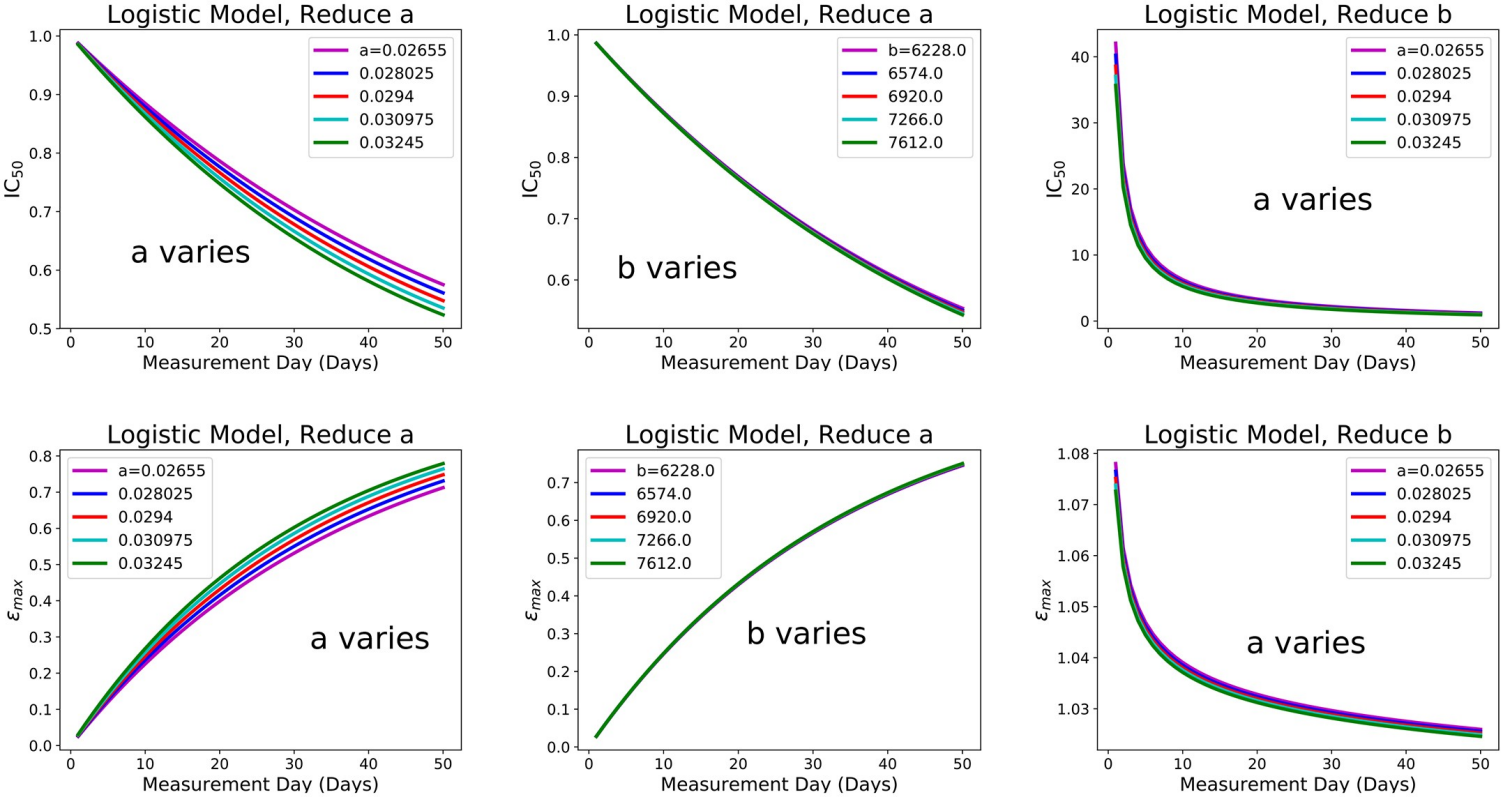
Sensitivity analysis for IC_50_ and *ε*_max_ as a function of measurement time. The base value of parameter *a* was varied in the first and last column, while parameter *b* was varied in the middle column for the logistic model with drug effect applied to parameter *a* (left and middle column) or drug effect applied to parameter *b* (right column).

In order to assess how our results depend on model parameters, we did a sensitivity analysis by varying model parameters and re-running the simulations. This allows us to assess how our results might change in different cell lines or patients, which are described by different parameter values [46], or to determine whether error in parameter estimates [47] will lead to large deviations in measured *ε*_max_ and IC_50_ values. Fig 3 shows how the *ε*_max_ and IC_50_ estimates change with changes in parameter values for the logistic model. The model parameters shown in the figures are the baseline, ± 5%, and ± 10%. Due to the large number of graphs created to have a complete sensitivity analysis for all seven models, only the sensitivity analysis for the logistic model for each type of variation for *ε*_max_ and IC_50_ is shown in this section. The remaining graphs are located in the supplementary material.

As seen in Fig 3, the smallest variation caused by changing parameter values occurs in the top middle graph when drug effect is applied to parameter *a* while parameter *b* is varied. We see the most variation when drug is applied to parameter *a* and the base value of *a* is itself varied. Most of the model predictions of estimated *ε*_max_ and IC_50_ values are not affected much by differences model parameters (supplemental material), with the Mendelsohn model showing the largest sensitivity to parameter values. Although even for this model, changes in parameter values did not change the general trend of decreasing IC_50_ and increasing *ε*_max_ values at later measurement times.

**Fig 4:**
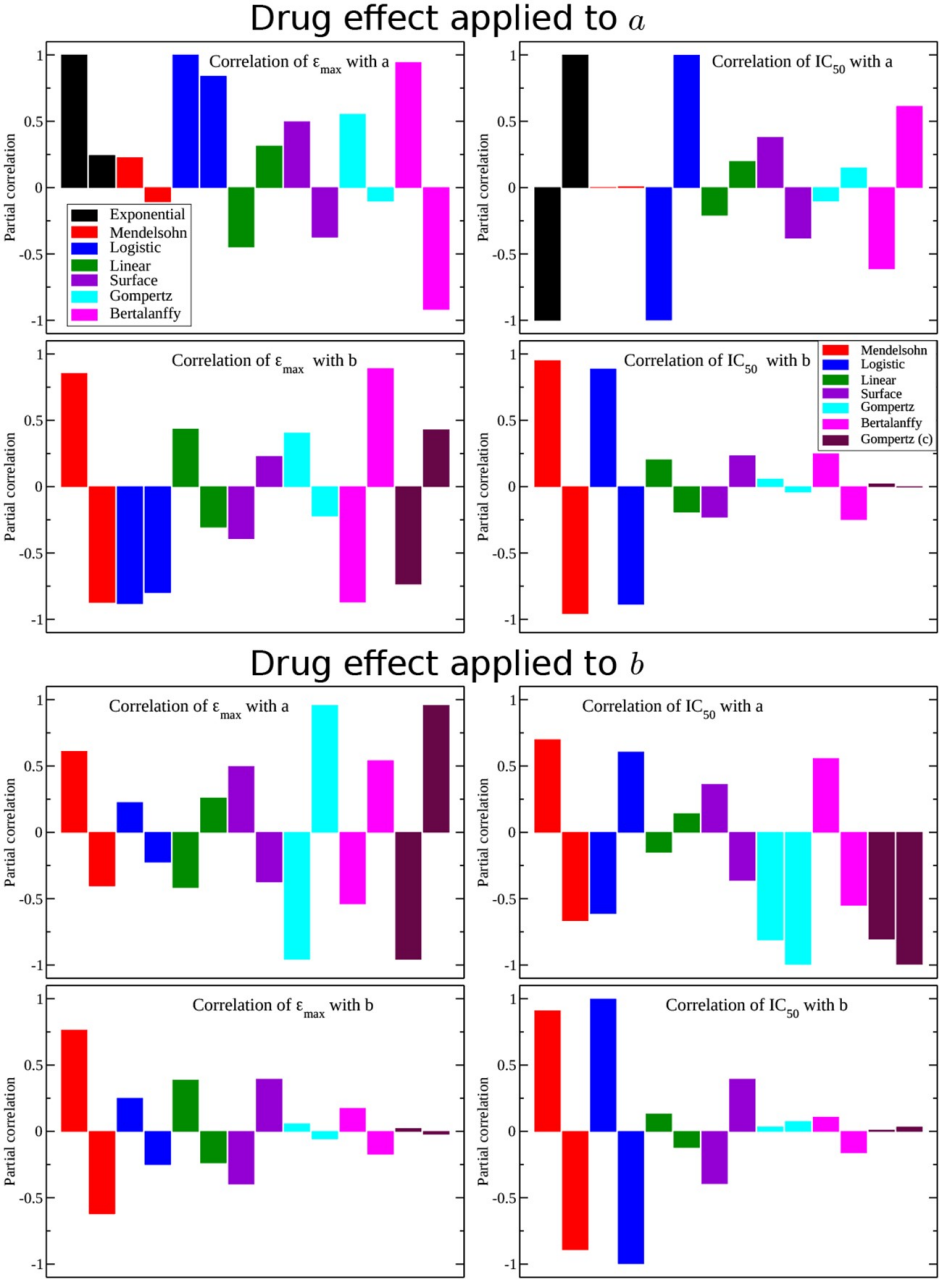
Partial correlation of *ε*_max_ (left column) and IC_50_ (right column) estimates at 10 days and 20 days (first and second bar of each color, respectively) on parameters *a* and *b* of each model. Note that we have included correlations of *ε*_max_ and IC_50_ with respect to the variable *c* in the Gompertz model in the maroon bars.

We also used the partial rank correlation coefficient to assess which models are most sensitive to changes in baseline parameters. We allowed parameter values to range between ±50% of their base value and used 1000 different randomly selected parameter combinations to calculate the partial rank correlation coefficient. Results are shown in Fig 4. The partial rank correlation coefficient is close to ±1 if there is a high degree of correlation between the independent and dependent variables. In our case, the independent variables are the model parameters *a*, *b*, *c* and the dependent parameters are *ε*_max_ and IC_50_ estimated at measurement days 10 and 20 (left and right bars of each color, respectively). The upper four models examine correlations when the drug effect is applied to parameter *a*, while the lower four panels examine the correlations when the drug effect is applied to parameter *b* (and *c* for the Gompertz model). Although there is no consistent trend, many models show opposite correlations for measurements taken at the two different times. For example, the Mendelsohn model shows a positive correlation between parameter values and measurements taken on day 10 (i.e. increasing *a* increases the estimated *ε*_max_ measured on day 10), but a negative correlation on day 20 (increasing *a* decreases the estimated *ε*_max_ measured on day 20). This indicates that in most cases, we do not see a simple upward or downward shift of the *ε*_max_ or IC_50_ vs. measurement time curve, but a change in the shape of the curve as parameter values change.

### Hill coefficient

**Fig 5:**
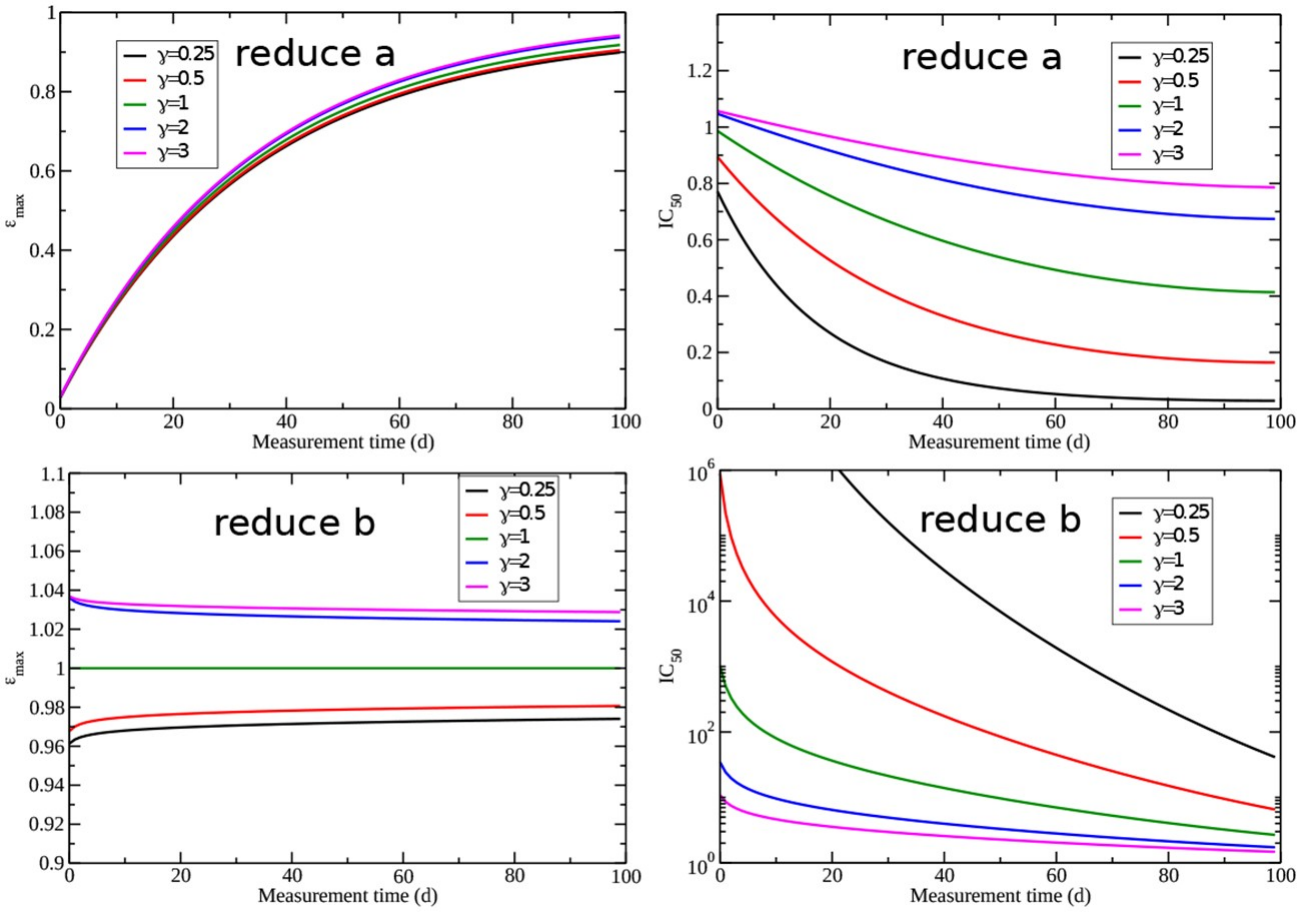
Effect of the Hill coefficient on estimates of *ε*_max_ (left column) and IC_50_ (right column) for the logistic model with drug applied to the growth rate *a* (top row) or the carrying capacity *b* (bottom row).

Although the Hill coefficient is often assumed to be 1 when incorporated into models, there is some experimental evidence that for chemotherapy drugs, the Hill coefficient can differ substantially from 1 [48]. While there have been only a handful of studies that incorporate the HIll coefficient for chemotherapy, there have been findings of Hill coefficients ranging from 0.3-3.0 [48–54]. We believe that it is useful to know how this coefficient alters our results for the measurements of drug characteristics (for both current and yet to be developed chemotherapeutic agents). Thus we also examined the role of the Hill coefficient on the estimates of drug efficacy parameters. Fig 5 shows the measurement time dependence of *ε*_max_ and IC_50_ for the logistic model with a drug that reduces the growth rate (top row) and a drug that reduces the carrying capacity (bottom row). While it might seem strange that a drug is modeled as reducing carrying capacity, which is traditionally thought to be determined by the tumor’s environment, there are therapies that modulate host factors and the tumor environment such as immune therapies [55] or kinase inhibitors [56, 57]. While a more appropriate model for these treatments would explicitly include the immune response [58, 59] or kinases [60], when using a simplistic model such as the logistic model, we could model the effect of such a therapy as limiting the capacity of the tumor to grow within the host.

Changing the Hill coefficient doesn’t have much effect on the measurement of *ε*_max_ for either assumption of drug effect. For a drug that reduces growth rate, increasing the Hill coefficient increases the measured values of IC_50_ making them closer to the real value. We see a very different effect if the drug is assumed to reduce the carrying capacity. In this case, the Hill coefficient can change the measured IC_50_ by several orders of magnitude. The effect of changing the Hill coefficient for the remaining models is shown in the supplementary material. All models predict that changes in the Hill coefficient have little effect on *ε*_max_ estimates, but can substantially change IC_50_ measurements.

## Discussion

This paper examined several commonly used ODE models of tumor growth and quantitatively assessed the differences in their predictions of quantities that characterize chemotherapy. We found that none of the models give the correct IC_50_ and *ε*_max_ values at any of the measurement times with the exception of the *ε*_max_ values when drugs reduce parameters *b* and *c* for the Gompertz model. Our sensitivity analysis indicates that both *ε*_max_ and IC_50_ predictions depend on model parameters. This is particularly problematic since there is error in parameter estimates when fitting models to data [46, 47] and this can exacerbate inaccuracy in estimates of *ε*_max_ and IC_50_ due to their dependence on measurement time.

IC_50_ in particular is used as a guide to help determine treatment dosages in patients [61, 62]. So we want a time independent measurement of IC_50_ because if IC_50_ depends on measurement time then the amount of drug used to treat patients could be incorrect. It is important to make sure the patient gets the right amount of drugs since chemotherapy drugs are toxic to both cancerous and non-cancerous cells. Many side effects from chemotherapy arise due to cancerous and non-cancerous cells being killed [63]. On the other hand, if a patient is not given enough drugs, then the tumor will continue to grow. Most of the models examined here predict that IC_50_ will be underestimated, with the discrepancy between measured and true IC_50_ increasing with increasing measurement time. This means that basing dosing recommendations on IC_50_ will most often lead to under-dosing of patients. Other models, however, suggest that the IC_50_ estimate can vary by orders of magnitude over only a few measurement days, particularly at early measurement times, leading to large variability in dosing recommendations. Other modeling studies have noted differences in IC_50_ values with measurement time [9, 12], with one finding a decrease in IC_50_ with measurement time [9] and the other finding that IC_50_ values increase with measurement time [12]. Experimental studies show more mixed results [9, 13–17], sometimes showing a decrease in IC_50_ with measurement time [9, 13–17], sometimes showing an increase in IC_50_ with measurement time [14, 15, 17], but also exhibiting more complex trends [13]. These discrepancies in model predictions indicate that much more work is needed to identify which models most accurately describe tumor growth [38, 64] since these growth models are increasingly being used to guide patient treatment decisions [65].

While *ε*_max_ is not commonly used to help determine treatment doses, we found that there is also a large variability in the estimates of this quantity as measurement time varies (up to 80% error). In line with the limited measurement of this quantity, we found only two previous studies of the time-dependence of *ε*_max_. One modeling study found that *ε*_max_ increases with measurement time [12], while an experimental study found that *ε*_max_ decreases with measurement time [13]. In our study, most of the growth models, however, suggest that later measurement times lead to more accurate estimates of *ε*_max_, suggesting a possible strategy here for getting more reliable estimates of *ε*_max_. Unfortunately, later measurement times often lead to larger errors in IC_50_, so there does not seem to be a way to measure both drug characteristics accurately using this measurement technique and new experimental techniques are needed. Given the importance of *ε*_max_ as a parameter defining the effectiveness of chemotherapy, there is clearly more need for studies to include estimates of this parameter to help us understand how best to measure *ε*_max_ and to help us understand the role it plays in determining the effectiveness of chemotherapy.

We also found that the Hill coefficient had only a small effect on estimates of *ε*_max_, but could have a larger impact on estimates of IC_50_. When drug effects are incorporated into models, the Hill coefficient is largely assumed to be one [66]. However, the limited number of studies that have attempted to measure the Hill coefficient for anti-cancer drugs have found values that range from 0.5 to 2.2 [48]. In order to incorporate mathematical models more effectively in patient treatment decisions, accurate measurements of the Hill coefficient need to be made for more drugs.

The models examined in this study are highly simplified and do not account for many factors that can affect measured IC_50_ and *ε*_max_ values in experiments. For example, we assume a constant dose of drug throughout the simulation. In reality, chemotherapy is not a continuous infusion, but is more accurately modeled by a time-dependent pharmacodynamic model [67]. Additionally, cancer cells are known to develop resistance to chemotherapy [68] which will also change measured IC_50_ and *ε*_max_ values. While some of these models assume spherical tumors, the models largely neglect detailed spatial structure [69] and tumor cell heterogeneity [70]. Our models are also not detailed enough to accurately capture the mechanisms of action of many chemotherapy agents [71]. These limitations are likely the reason there is discrepancy between our findings and experimental measurements [13–17]. However, the models examined here form the basis for many more complex tumor growth models [72–75], so their behavior needs to be understood in detail.

Overall, our results indicate that better measurement techniques are needed to estimate IC_50_ and *ε*_max_. Already, some studies have started investigating other possible measures to characterize drug effect. Fallahi-Sichani et al. fallahi13 suggest using additional features of the dose-response curve, such as the area under the curve (AUC) or the slope of the curve, for a more complete characterization of the effect of a drug. Calhelha et al. [76] also suggest using other points on the dose response curve such as the minimal response or the dose with the maximum affinity in the response reaction. Unfortunately, these suggestions still uses dose-response curves generated by measuring number of cells (or tumor volume) on a particular day and these additional measures might suffer from the same time-dependence problem as IC_50_ and *ε*_max_. A series of other studies have suggested using growth rate (GR) to generate dose-response curves [9, 10, 12]. Growth rate is largely independent of measurement time after a short transient phase and before the plateau [9]. Thus, dose-response curves can be generated that result in consistent GR_50_ and GR_*max*_ estimates [12]. While this is a promising idea, there are still problems with implementing the technique in a manner that is reproducible in different research centers [10].

## Conclusion

Our results show that IC_50_ and *ε*_max_ are time-dependent values and are also sensitive to model choice and model parameters, making it difficult to accurately estimate these parameters experimentally. It is our hope that our work will spur more investigation into better methods to determine the correct doses before testing drugs in patients to get the most effective therapeutic treatment.

## Supporting information

S1 File Supplemental information. Pdf file containing additional figures for parameter sensitivity analysis and Hill coefficient dependence.
(PDF)
