# Supplementary material for: Understanding the effect of measurement time on drug characterization
Source: PLoS One. 2020 May 14;15(5):e0233031. doi: 10.1371/journal.pone.0233031 (PMC7224495; doi:10.1371/journal.pone.0233031)
Supplement: S1 File — Pdf file containing additional figures for parameter sensitivity analysis and Hill coefficient dependence. (PDF) [file pone.0233031.s001.pdf]

Supplement for “Understanding the Effect of Measurement Time on Drug Characterization”

Hope Murphy Gabriel McCarthy Hana M. Dobrovolny

October 19, 2019

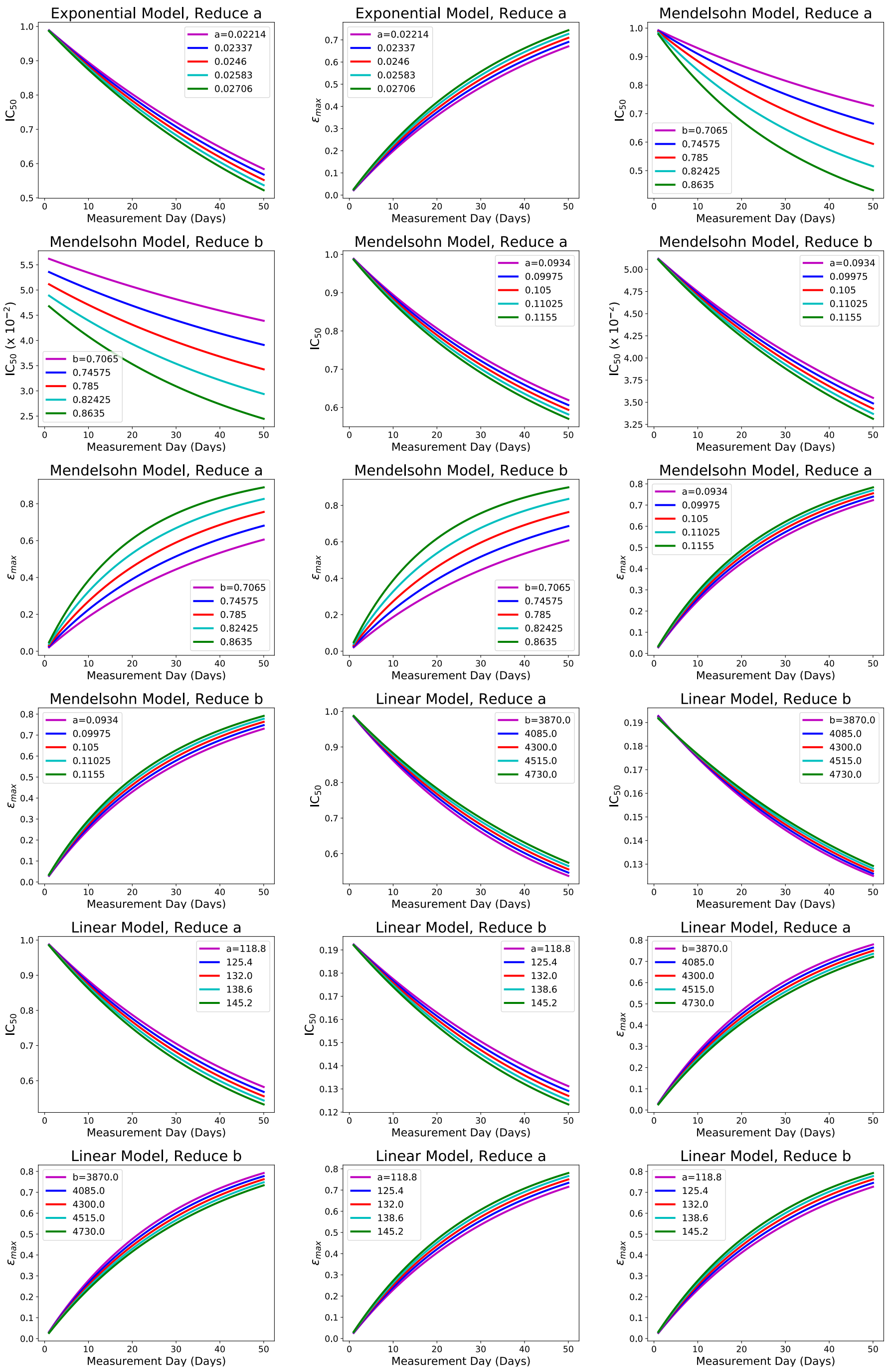

Figure 1: Sensitivity of $\varepsilon_{\max}$ and IC_{50} time-dependence on model parameters.

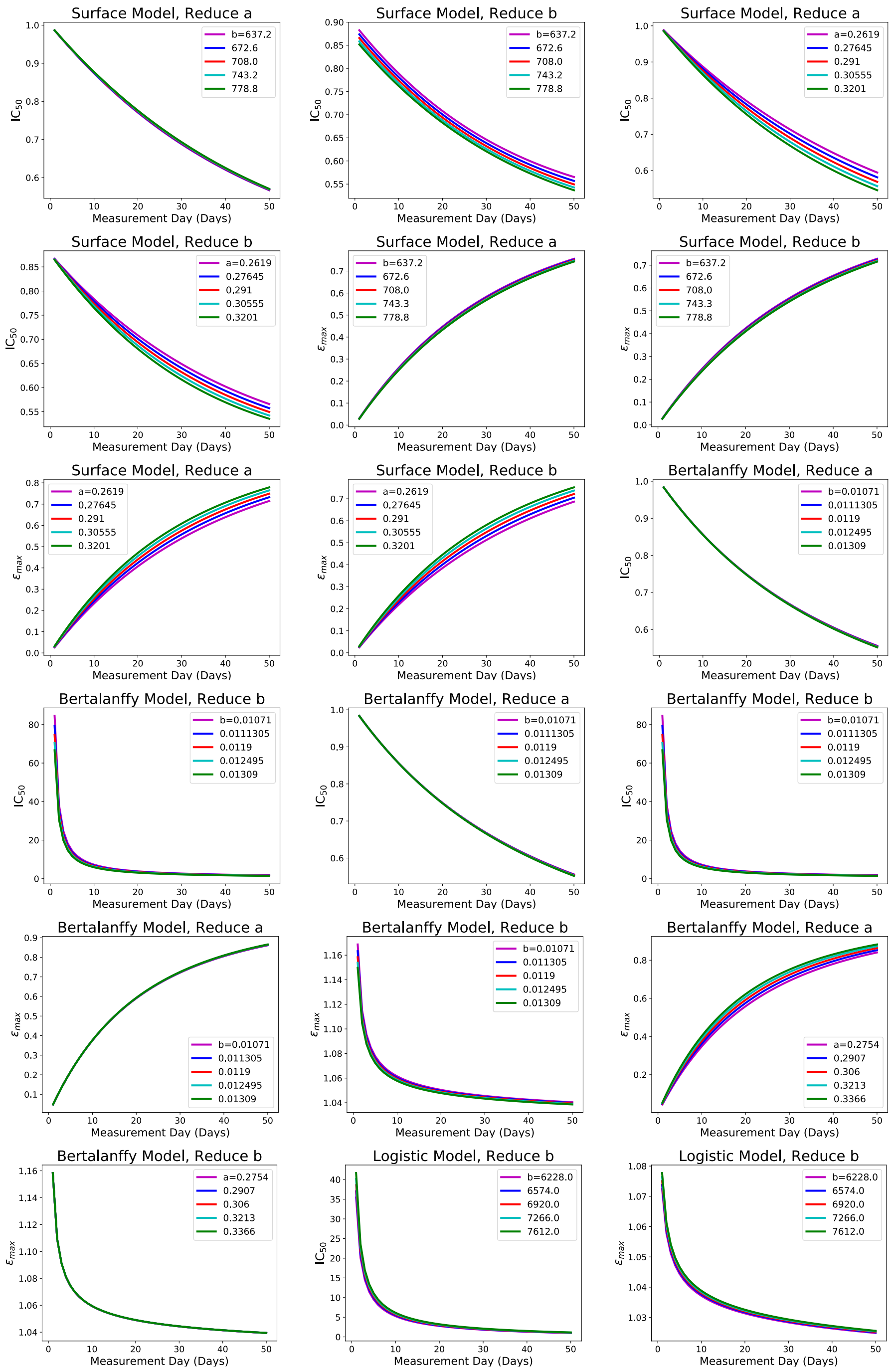

Figure 2: Sensitivity of $\varepsilon_{\max}$ and IC_{50} time-dependence on model parameters.

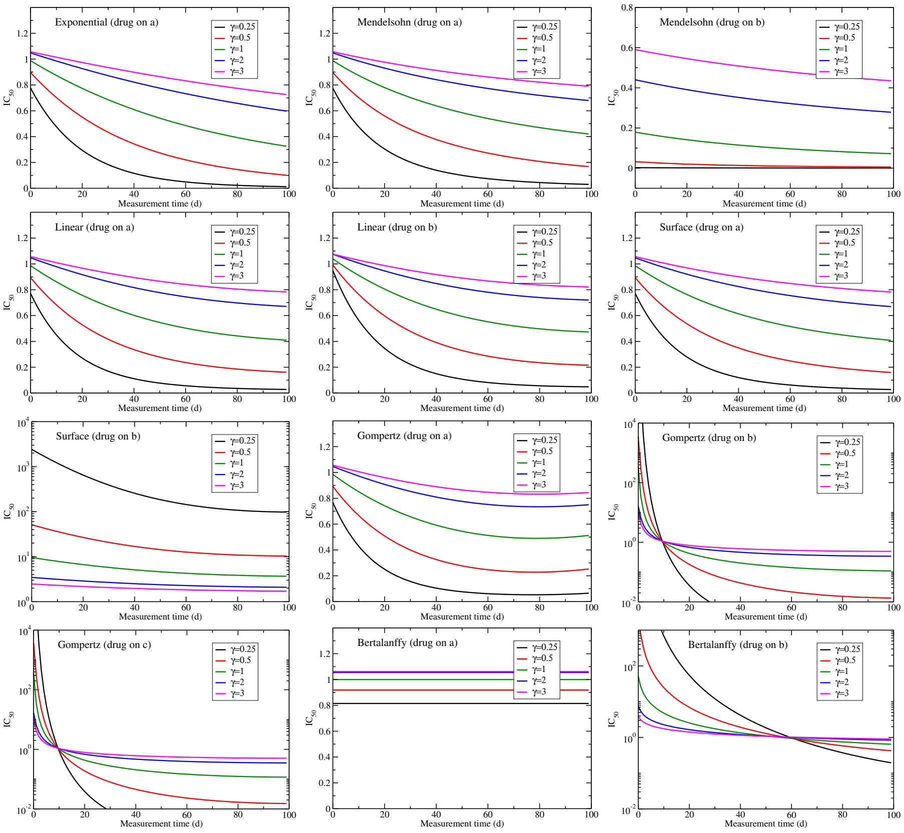

Figure 3: Dependence of IC_{50} time-dependence on the Hill coefficient.

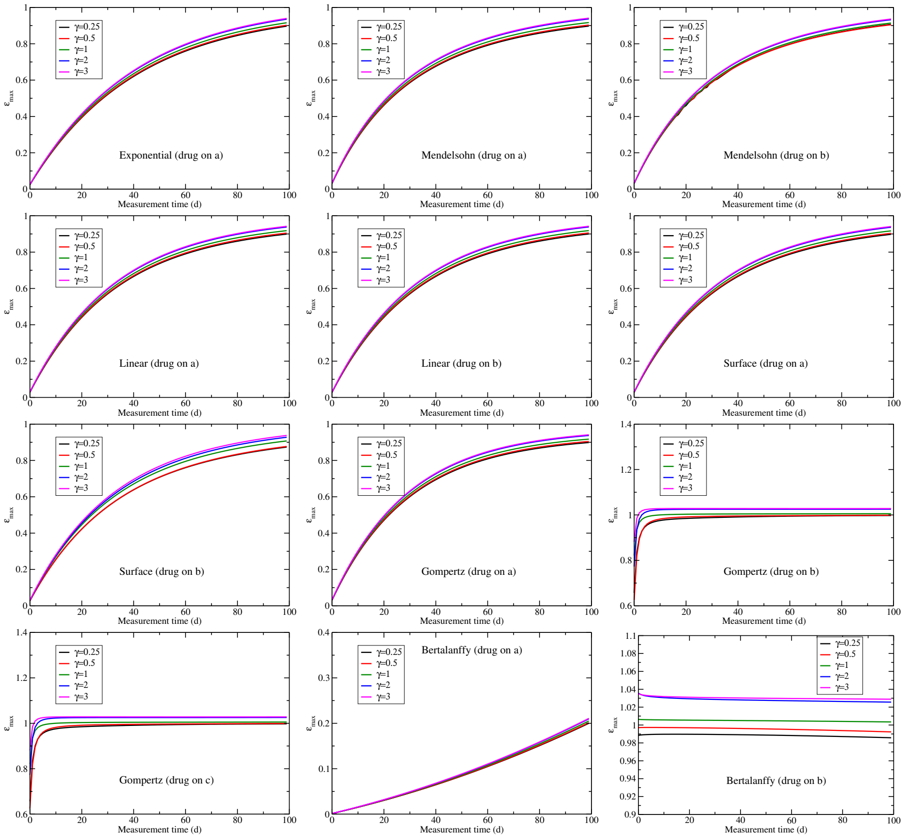

Figure 4: Dependence of $\varepsilon_{\max}$ time-dependence on the Hill coefficient.
